# Phylogenetic Analysis of Wesselsbron Virus Isolated from Field-Captured Mosquitoes during a Rift Valley Fever Outbreak in Kabale District, Uganda—2016

**DOI:** 10.4269/ajtmh.22-0481

**Published:** 2022-11-21

**Authors:** John T. Kayiwa, Martin N. Mayanja, Teddy Muwawu Nakayiki, Fred Senfuka, Julius Mugga, Jeffrey W. Koehler, Eric C. Mossel, Julius J. Lutwama

**Affiliations:** ^1^ Department of Arbovirology, Emerging, and Re-emerging Diseases, Uganda Virus Research Institute, Entebbe, Uganda;; ^2^ U.S. Army Medical Research Institute of Infectious Diseases, Frederick, Maryland;; ^3^ Division of Vector-Borne Diseases, U.S. Centers for Disease Control and Prevention, Fort Collins, Colorado

## Abstract

After confirmation of two human cases of Rift Valley fever (RVF) in March 2016 in the Kabale district of Uganda, an entomological investigation was conducted with a focus on mosquito species composition and abundance of known and potential mosquito vector species, and virus testing to identify species most likely involved in Rift Valley fever virus transmission. This information could be used to forecast risk and facilitate improvement of prevention and response tools for use in preventing or controlling future outbreaks. From these collections, two virus isolates were obtained, one each from a pool of *Aedes tricholabis* and *Ae. gibbinsi*. Next-generation sequencing identified both isolates as Wesselsbron virus, family *Flaviviridae*, a neglected arbovirus of economic importance. These are the first reported Wesselsbron virus isolates from Uganda since 1966.

Wesselsbron virus (WESSV) is a mosquito-borne virus of the family *Flaviviridae,* genus *Flavivirus*, that is widely distributed in Africa. WESSV infection causes Wesselsbron disease in sheep and cattle. The virus was first isolated in 1955 in South Africa from the brain of a dead lamb during an outbreak in Wesselsbron District in the Free State Province and from a febrile human and mosquitoes in the Natal Province.^1^^,^^2^ In sheep, the disease is clinically similar to Rift Valley fever (RVF), which is caused by RVF virus (RVFV) infection and often results in abortions and 20% mortality in pregnant ewes.^3^ Outbreaks of WESSV may go unnoticed because they are often concomitant with RVF epidemics,^2^ and therefore the incidence may be underestimated. In humans, infection typically presents as influenza-like illness causing arthralgia, myalgia, and fever during a short and mild acute phase.^4^ However, occupational exposure via the ocular mucosa occurred in a laboratory worker resulting in neurological symptoms, including intense headache, slurred and repetitive speech, severe muscle spasms, and memory loss, with fever of 40°C, abdominal pain, and liver tenderness. Complete symptom resolution took several weeks with memory loss and joint pain lasting a particularly long time.^5^

In addition to humans, WESSV has been isolated from several livestock species, including camels, cattle, pigs, donkeys, sheep, and horses, and serological evidence of infection has been found in small mammals, dogs, nonhuman primates, birds, and wild ruminants.^4^ WESSV has been isolated from mosquitoes collected in South Africa, Botswana, Zimbabwe, Uganda, Mozambique, Cameroon, Central African Republic, Mauritania, Senegal, Nigeria, Democratic Republic of the Congo, and Madagascar.^6^ The virus is most frequently associated with *Aedes* species mosquitoes, with a few isolations reported from *Culex* (*Culex*), *Mansonia* (*Mansonioides*), and *Anopheles* (*Celia*) mosquitoes and one isolation reported from an Ixodid tick. *Aedes* subgenera from which WESSV has been isolated tend to follow a geographic pattern with *Ae*. (*Neomelanoconion*) predominating in southern Africa, *Ae.* (*Aedimorphus*) in East Africa, and both *Ae.* (*Adm*) and *Ae*. (*Stegomyia*) in West Africa, with reported isolations from several species in each subgenus.^4^

The only reported isolation of WESSV in Uganda occurred in 1966 from a pool of unspeciated *Ae*. (*Adm*) mosquitoes collected in the West Nile District (now the West Nile subregion) of northwest Uganda (Figure 1).^7^ Here we report the isolation and genomic characterization of WESSV by the arbovirus laboratory at the Uganda Virus Research Institute, from mosquitoes collected in Kabale District in southwest Uganda in 2016, after the laboratory confirmation of two human cases of RVF.^8^ After confirmation of the RVF cases, an entomological investigation was performed in Kabale District to determine the epidemic/epizootic vectors of RVFV. The entomological surveillance focused on factors contributing to the epidemic—specifically, mosquito species composition, abundance of known and potential mosquito vector species, and virus testing to identify species most likely involved in virus transmission in the affected areas. The overall goal was to learn more about the vectors involved in RVFV maintenance and transmission during outbreaks to generate information that could be used to forecast risk and facilitate improvement of prevention and response tools for use in preventing or controlling future outbreaks.

**Figure 1.**
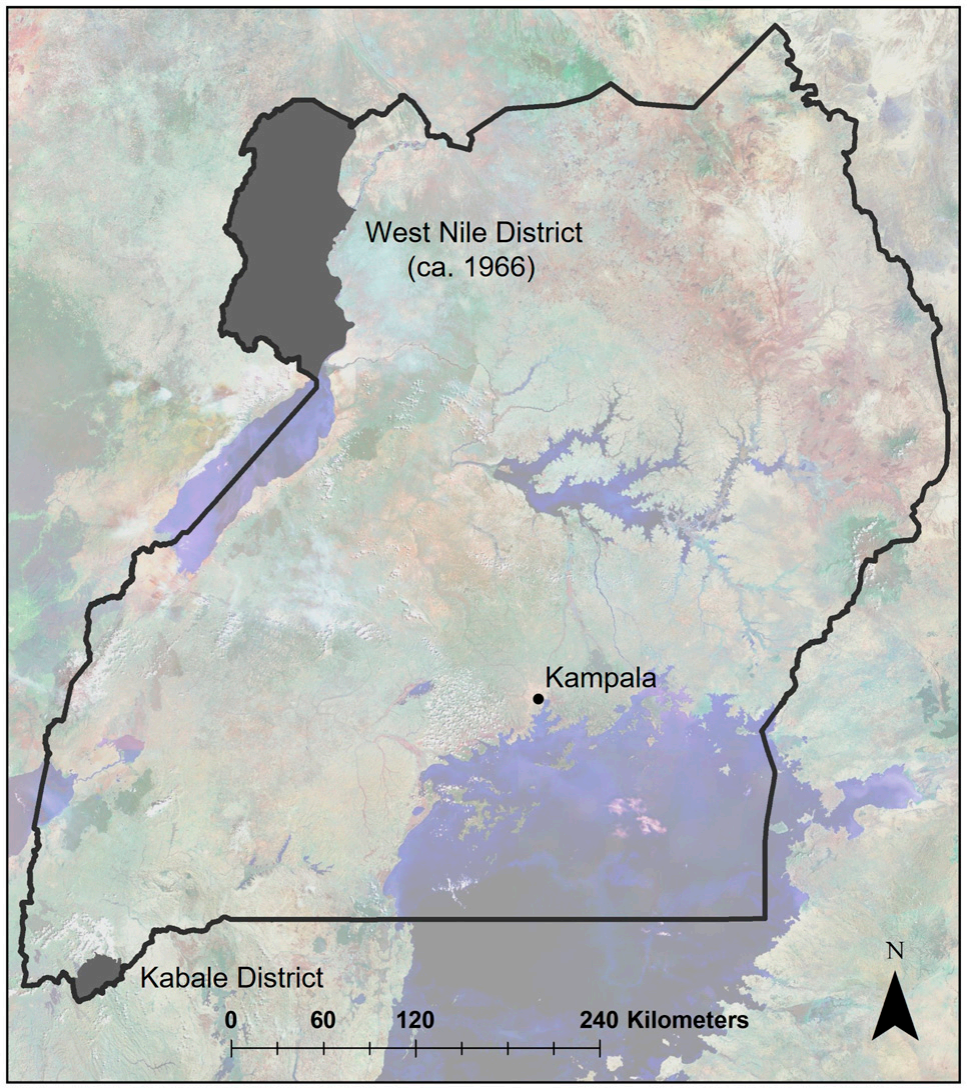
Map showing the locations of Wesselsbron virus (WESSV) isolations in Uganda. The West Nile District in 1966 (shaded, upper left), now constituting most of the West Nile subregion, was the collection location of the mosquitoes yielding the original WESSV isolation in Uganda. The current isolate was obtained from mosquitoes in Kabale District in 2016 (shaded, lower left) during an entomological investigation after a Rift Valley fever outbreak.

Mosquitoes were collected from five sites in areas where confirmed or suspected RVF cases were previously reported.^9^ The mosquitoes were sampled using CDC miniature light traps (Clarke Mosquito Control, Roselle, IL) with dry ice as a source of carbon dioxide and an attractant as previously described.^10^ Mosquitoes trapped in overnight collections were immobilized by freezing on dry ice for a few minutes, immediately counted, and placed in cryotubes. Cryotubes in boxes were transported in liquid nitrogen dry shippers to the arbovirus laboratory at the Uganda Virus Research Institute. There the mosquitoes were identified on ice to species based on morphological characters using various taxonomic keys as described previously.^10^ Identified mosquitoes were pooled (≤ 25 mosquitoes per pool) by species, sex, gravid or engorged status, collection date, and site and frozen at −80°C for later testing.

A total of 298 pools (9,950 mosquitoes representing 33 species from six genera) were collected from five locations: Bugongi village near the home of the first confirmed human case, Kamuganguzi subcounty near the home of the second confirmed human case, Rubaya subcounty in the home of a probable human case, Kitumba subcounty near Kabale town council, and in Bubaale subcounty near Lake Bunyoni.^9^

The mosquito pools were homogenized and clarified by centrifugation in 2-mL conical microcentrifuge tubes.^9^ Clarified homogenates were immediately processed further or stored at −80°C. All mosquito supernatants were first tested for RVFV by reverse transcription polymerase chain reaction (RT-PCR).^9^ Negative RVFV supernatants were then tested for other infectious viruses by inoculating 100 µL on Vero cells in six-well plates and overlaid with nutrient agarose in a modified plaque assay.^11^ Plates were overlaid a second time with nutrient agarose containing neutral red stain 4 days after inoculation and followed for up to 10 additional days to observe for plaque formation. Two wells, one each from homogenates of *Ae.* (*Adm*) *tricholabis* (pool 5215) and *Ae.* (*Adm*) *gibbinsi* (pool 5236) exhibited plaques on day 4 post-inoculation. These wells were harvested on day 6 by removing the agarose plug and washing the monolayer with 0.5 mL BA-1 medium.^12^ Putative viruses were amplified by infecting Vero cells in 25-cm^2^ flasks with 100 µL of plaque isolate.^13^ Flasks were observed for cytopathic effects and supernatants harvested on day 6 after inoculation.

RNA was extracted from cell culture supernatants (QIAamp Viral RNA Mini Kit, Qiagen, Inc., Germantown, MD) and identification of virus isolates was attempted by RT-PCR using arbovirus group-specific primers for alphaviruses, orthobunyaviruses, and flaviviruses.^14^^–^^17^ RT-PCR amplification of the RNA from the two supernatants determined both were flaviviruses. RT-PCR testing using virus-specific primers for yellow fever virus, West Nile virus, Zika virus, and dengue virus 1–4 was negative.

To identify the viruses using next-generation sequencing, total nucleic acid was extracted from 100 µL supernatant using 300 µL TRIzol LS (Thermo Fisher Scientific, Waltham, MA) and the EZ1 XL Advanced with the EZ1 Virus Mini kit 2.0 (Qiagen, Valencia, CA). Each sample was eluted in 60 µL elution buffer and amplified using the SeqPlex WTA kit (Sigma-Aldrich, St. Louis, MO). Libraries generated using the Apollo 324 System (WaferGen Biosystems Inc., Fremont, CA) using TruSeq HT adapters (Illumina, San Diego, CA) and the PrepX ILM 32i DNA Library Kit (WaferGen Biosystems Inc.) were sequenced on the MiSeq platform (Illumina) using the MiSeq Reagent Kit v2 cycle 300.

All sequence assembly and analysis was conducted using CLC Genomics Workbench v. 10.1.2 (Qiagen). Sequence reads were trimmed for quality and filtered to remove reads mapping to coliphage phi-X174 (NC_001422.1) and the human H19 reference genome (NC_000001–NC_000024). The remaining reads were de novo assembled (at least 70% read length with 80% similarity) and BLAST analyzed for identification. A draft consensus genome was generated by mapping the trimmed reads to the top BLAST hit. Mapping the trimmed reads back to this draft consensus sequence generated a final/consensus sequence. Sequence alignments and phylogenetic trees (Neighbor-Joining with Jukes-Cantor and 1,000 bootstrap replicates) were generated using the final draft sequences generated here and all near full-length sequences for each WESSV isolate available in GenBank (accessed March 31, 2022) and MEGA version X.^18^

The RNA isolated from the two virus cultures yielded de novo contigs that were BLAST-identified as Wesselsbron virus (WESSV), with the closest sequence identity being an isolate collected from South Africa in 1997 (Figure 2). A pairwise comparison of the two WESSV genomes from Uganda showed 97.5% identity between the two isolates and 94.4% to 96.5% identity with the next closest known sequence in GenBank (AV259/ZAF/1997; accession number: JN26796).

**Figure 2.**
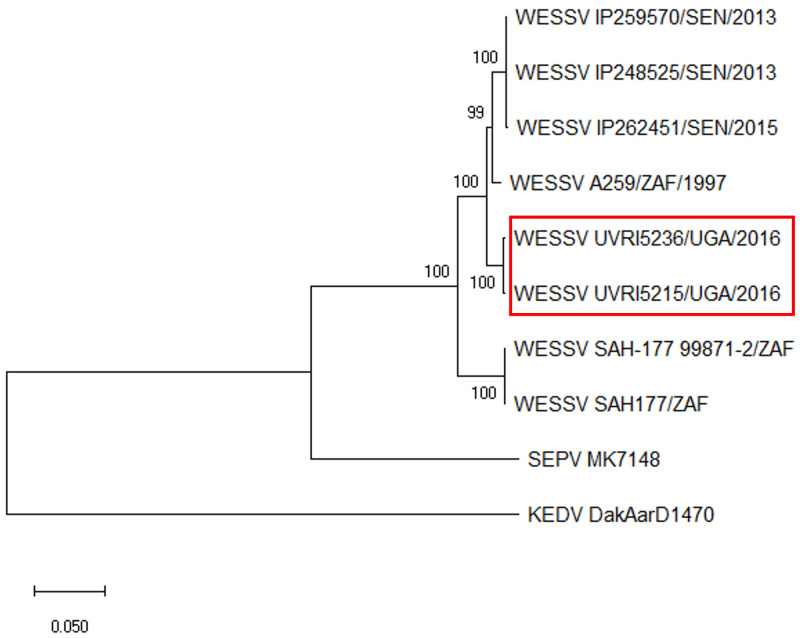
Phylogenetic analysis of new Wesselsbron virus (WESSV) isolates. Near full-length WESSV sequences in GenBank were aligned, and a phylogenetic tree (neighbor joining with Jukes-Cantor and 1,000 bootstrap replicated) was generated using MEGA version X. Isolates described in this article are boxed. Specific source countries are indicated: South Africa (ZAF), Uganda (UGA), and Senegal (SEN). GenBank accession numbers for listed isolates from top to bottom: KY056258, KY056256, KY056257, JN26796, **ON157055**, **ON157054**, DQ859058, NC_012735, DQ837642, and NC_012533. Accession numbers of isolates identified herein are bold.

Historically, certain arboviruses have been extensively studied under the rubric of emerging and reemerging diseases or biodefense pathogens, and as a result, a great deal of knowledge about their molecular biology, pathogenesis, and potential to reemerge as public health threats at a global scale is known. Of note, most arboviral infections and disease consequences have been neglected, including WESSV. Arboviruses more severely affect impoverished individuals and promote poverty in endemic regions by directly and indirectly causing long-lasting and devastating effects on the quality of life. For example, RVFV is embedded in the ecosystems of many poor African countries, and during outbreaks or epizootics disproportionately attack the health and family incomes of seminomadic pastoralists.^19^ Furthermore, most mosquitoes carrying arboviruses are not anopheline species, which live peridomestically and feed at night. These nonanopheline mosquitoes feed during the day or at dusk, both outdoors and indoors, increasing the risk to humans and livestock and limiting the utility of measures such as mosquito nets. In this investigation, an abundance and diversity of mosquito vectors was demonstrated in Kabale District,^9^ which is likely to create variable points of risk for livestock exposure to arboviral disease and subsequent human disease occurrence.

We report the first isolation of WESSV in Uganda in 50 years and the first isolation from field-collected mosquitoes identified to the species level, notably *Ae. tricholabis* and *Ae. gibbinsi*. Given the relatively few complete genome sequences in GenBank for WESSV (*N* = 6), this additional genetic information will help guide assay development efforts for this important but neglected virus.

Although no fatal human cases have been identified, the impact of WESSV on public health may be underestimated given the lack of monitoring of the virus. In fact, most human cases have been identified in the context of other investigations in which the described human cases of WESSV were found to be coinfections. Therefore, further investigations are needed in human populations, arthropod vectors, and mammals to understand the life cycle and the impact of WESSV on public health.
